# Enhanced biodegradation of phenanthrene and anthracene using a microalgal-bacterial consortium

**DOI:** 10.3389/fmicb.2023.1227210

**Published:** 2023-09-13

**Authors:** Mubasher Zahir Hoque, Abdulrahman Alqahtani, Saravanan Sankaran, Deepak Anand, Musa M. Musa, Alexis Nzila, Gea Guerriero, Khawar Sohail Siddiqui, Irshad Ahmad

**Affiliations:** ^1^Department of Bioengineering, King Fahd University of Petroleum and Minerals, Dhahran, Saudi Arabia; ^2^Department of Chemistry, King Fahd University of Petroleum and Minerals, Dhahran, Saudi Arabia; ^3^Interdisciplinary Research Center for Refining and Advanced Chemicals, King Fahd University of Petroleum and Minerals, Dhahran, Saudi Arabia; ^4^Interdisciplinary Research Center for Membranes and Water Security, King Fahd University of Petroleum and Minerals, Dhahran, Saudi Arabia; ^5^Environmental Research and Innovation (ERIN) Department, Luxembourg Institute of Science and Technology, Hautcharage, Luxembourg; ^6^School of Biotechnology and Biomolecular Sciences (BABS), The University of New South Wales, Sydney, NSW, Australia

**Keywords:** microalgal-bacterial consortium, polycyclic aromatic hydrocarbons, phenanthrene and anthracene, biodegradation genes, petrogenic pollutants, water contaminants, *Gonium pectorale*

## Abstract

Polycyclic aromatic hydrocarbons (PAHs) are chemicals that are released into the environment during activities of the petroleum industry. The bioaccumulation, carcinogenic and mutagenic potential of PAHs necessitates the bioremediation of these contaminants. However, bioremediation of PAHs has a number of limitations including the inability of a single microbe to degrade all of the PAH fraction’s environmental constituents. Therefore, a different paradigm, employing microalgal-bacterial consortium (MBC), may be used to effectively remove PAHs contaminants. In this type of interaction, the microalgae and bacteria species in the consortium work together in a way that enhances the overall performance of the MBC. Bacterial species in the consortium provide essential nutrients or growth factors by degrading toxic substances and provide these to microalgae, while the microalgae species provide organic carbon for the bacterial species to grow. For the first time, the ability of *Gonium pectorale* (*G. pectorale*) microalgae to break down phenanthrene (PHE) and anthracene (ANT) was investigated. Phenanthrene was shown to be more effectively degraded by *G. pectorale* (98%) as compared to *Bacillus licheniformis* (*B. licheniformis*) 19%. Similarly, *G. pectorale* has effectively degrade anthracene (98%) as compared with *B. licheniformis* (45%). The consortia of *G. pectorale* and *B. licheniformis* has shown a slight increase in the degradation of PHE (96%) and ANT (99%). Our findings show that *B. licheniformis* did not inhibit the growth of *G. pectorale* and in the consortia has effectively eliminated the PAHs from the media. Therefore *G. pectorale* has a tremendous potential to remove PAHs from the polluted environment. Future research will be conducted to assess *Gonium*’s capacity to eliminate PAHs that exhibit high molar masses than that of PHE and ANT.

## Introduction

Across recent decades, environmental pollution has been a global problem of great concern due to its detrimental effects on both human health and ecosystems. Anthropogenic activities have discharged a significant amount of pollutants into the environment, and the petroleum sector produces vast amounts of crude oil wastewater with significant pollution (Mojiri et al., 2019; Olajire, 2020; Alvarez-Ospina et al., 2021). Numerous investigations have shown that toxic substances, such as aliphatic or aromatic hydrocarbons, phenolic compounds, heavy metals, their derivatives, and chemicals used in the de-emulsification process, are always present in crude oil discharge (Trannum et al., 2011; Uzoekwe and Oghosanine, 2011). The repercussions for living things of the buildup of such substances in the associated water bodies are severe (Ngwoke et al., 2021).

Polycyclic aromatic hydrocarbons (PAHs), which have two or more fused aromatic rings arranged angularly, linearly, or in clusters, are regarded as a fundamental component of petroleum sewage. They are discovered in crude oil, which is created through the burning of fuel, the burning of waste, or as a byproduct of industrial activities like petroleum exploration (Suman et al., 2016; Lawal, 2017). Given their water solubility, toxicity, bioaccumulation potential, and persistent character, PAHs have been referred to as the main environmental pollutants that pose the greatest health risks (Dong et al., 2012; Adeniji et al., 2019; Patel et al., 2020). Various physicochemical, thermal, and photolytic remediation techniques are used; however, they have drawbacks such as the use of a high chemical dosage, energy demand, high investment, massive operating costs and cannot entirely rid the environment of toxins from the polluted site along with their negative effect on the environment (e.g., spent washing solution, air pollution, flora destruction). Hence, bioremediation with microorganisms is a sustainable, socially acceptable, and environmentally benign alternative to traditional remediation techniques that helps to restore the environment (Abdel-Shafy and Mansour, 2016; Gupte et al., 2016; Qutob et al., 2022). The sequence of decreasing susceptibility is followed by the biodegradability of petroleum hydrocarbons: linear alkanes > branched alkanes > monoaromatics > cyclic alkanes > polyaromatics > asphaltenes (Varjani and Upasani, 2017).

Bacteria often existed in a variety of habitats and were uniquely metabolically adaptable to break down PAHs in both aerobic and anaerobic environments. O_2_, which serves as both a co-substrate for the hydroxylation process involving the monooxygenase and dioxygenase enzymes and an absolute electron acceptor in aerobic conditions, breaks down an aromatic ring of a PAH molecule (Chen et al., 2016). While bacteria use an altogether different strategy to break down an aromatic ring in the absence of oxygen, subjecting it to reductases and other final electron acceptors. In ecosystems like phreatic zone, deep sea sediment, and water-flooded soil, anaerobic breakdown of PAHs is more successful (Ghosal et al., 2016; Dhar et al., 2020). PAHs are naturally broken down by bacteria to provide them with the carbon they need for energy. However, the cytotoxic effects of petroleum hydrocarbons, poor climatic circumstances, longer time, metabolic restrictions, hydrocarbon composition, and concentration inhibit its capacity to degrade (Xu et al., 2018).

The importance of microalgae, which are photosynthetic microorganisms present in terrestrial and marine ecosystems, has increased due to their widespread distribution, ease of cultivation, and exceptional capacity to degrade PAHs. The degradation of PAHs by microalgae has been documented in certain research at the laboratory or microcosm size, but field-scale PAH cleanup requires the development and optimization of advanced techniques. Several microalgae strains have been found to breakdown PAHs such as benzo[a]pyrene, phenanthrene, anthracene, and naphthalene (de Llasera et al., 2016, 2018).

For the biodegradation of resistant PAHs, researchers are currently concentrating on microalgae bacterium consortia. This co-culturing method, which combines bacteria and microalgae, is more advantageous than employing just one species and is thus more promising (Ahmad, 2021; García et al., 2021). The organisms coexist in a symbiotic relationship in a microalgae bacteria consortium, where microalgae provide oxygen during photosynthesis that may promote bacterial development. Furthermore, produced for the growth of microalgae are several vital vitamins, minerals, and trace elements, as well as crucial phytohormones, chelating agents, and other substances (Wang et al., 2016). Moreover, the bacteria may have a stable environment to grow on the microalgae cell surface, while pollutants present there may create a pollutant-augmented zone that will hasten the degradation of PAHs (Gutierrez et al., 2014). The greatest method to increase the degradation capability of these organisms in the removal of persistent PAHs from the contaminated ecosystems in the biosphere may be the symbiotic association between microalgae and bacteria (Kleindienst et al., 2015; Perera et al., 2018; Yao et al., 2018).

To the best of our knowledge, the symbiotic association between microalgae and bacteria has never been documented in the literature. The goals of this study were to (1) test the ability of green microalgae (*G. pectorale*) and bacteria (*B. licheniformis*) to grow together in the same media, and (2) determine the PAHs (phenanthrene and anthracene) degradation potential of microalgae and bacteria separately and in consortia settings.

## Methods and materials

### Culture media and microorganisms used in this study

*G. pectorale* obtained from the Microbial Culture Collection at the National Institute for Environmental Studies, Tsukuba, Japan; Available online: http://mcc.nies.go.jp/ was grown under continuous light (1,300 lux) in 50 mL of modified Bold’s 3 N medium adjusted to pH 6.8 (Guerriero et al., 2018). *B. licheniformis* isolated from petroleum contaminated soil in a previous study (Nzila et al., 2019) was used to test its compatibility with *G. pectorale*. For reviving bacterial cultures, a loop-full of bacteria from stock plates were transferred to nutrient broth and grown overnight at 37°C in a rotary shaker (Patel et al., 2015; Kahla et al., 2021).

*G. pectorale* was grown for a month in one-liter B3N in two 2-L flasks (total 2.0 L algae culture from both flasks combined). After 30 days, algae from both flasks were concentrated to 20 mL by centrifugation. This concentrated solution was then utilized in the main experiments. Bacteria were grown in 200 mL NB overnight whenever needed. Bacterial solution was always concentrated and adjusted to OD 1.0 for all experiments involving bacteria.

### PAH preparation

The ability of the microbial strains to degrade phenanthrene (PHE) and anthracene (ANT) was tested in this experiment. Stock solutions of the PAHs were prepared by separately dissolving 0.1 g of each of the PAHs in 10 mL dimethyl sulfoxide (DMSO) to make a final concentration of 10,000 ppm. Next, working concentration of 5 ppm (5 mg/L) for the experiments were calculated using the formula C_1_V_1_ = C_2_V_2_ (Nzila et al., 2018).

### Algae-bacteria growth compatibility experiments

For compatibility experiments, 200 mL of B3N medium adjusted to pH 6.8 was dispensed aseptically into each 500 milliliters Erlenmeyer and prepared as follows (Table 1).

**Table 1:** Summary of the culture experiments performed in this study, G = *Gonium*, B = *Bacillus*.

Experiment title	*G. pectorale*	*B. licheniformis*	PHE	ANT
G-only	✓	✘	✘	✘
B-only	✘	✓	✘	✘
GB-only	✓	✓	✘	✘
G-PHE	✓	✘	✓	✘
B-PHE	✘	✓	✓	✘
GB-PHE	✓	✓	✓	✘
G-ANT	✓	✘	✘	✓
B-ANT	✘	✓	✘	✓
GB-ANT	✓	✓	✘	✓

Next, 1.0 mL suspension of isolates in a ratio of 1:1 for microalgae and bacteria was aseptically dispensed into the appropriate flasks. Culture temperature was maintained at 30°C in a rotary shaker at 170 rpm. All cultures received light from a white LED source (1,300 lux) at a light: dark ratio of 15:9 h/day. Experiments were conducted for 30 days.

To monitor growth of *G. pectorale*, 20.0 mL of culture media was removed weekly from algae cultures and filtered for dry weight measurement. Cultures were removed from flasks using sterile disposable 10 mL pipettes. Weight (W1) of dry (empty) filter papers were recorded prior to usage. After filtering, the filter papers with the *G. pectorale* were allowed to dry before measuring the weight again (W2). The weight of *G. pectorale* was then calculated (dry weight of algae = W2 – W1) as described by Ratha et al. (2016).

Bacterial growth was measured with plate count technique every 3 days. Bacterial cultures (1.0 mL) were removed from their respective flasks and serially diluted. Diluted cultures were then streaked onto nutrient agar plates and kept overnight at 37°C. The following day, colonies were counted on plates having 30–300 colonies per plate. Next, the CFU/ml was calculated for the original samples to generate growth curves (Nzila et al., 2021).

### PAH degradability experiments

For degradability experiments, 50.0 mL of B3N medium adjusted to pH 6.8 was dispensed aseptically into each 250-ml Erlenmeyer flasks and prepared as reported previously with some modification (Nzila et al., 2021) (Table 2).

**Table 2:** Summary of the PAHs degradability experiments performed in this study.

Experiment title	*G. pectorale*	*B. licheniformis*	PHE	ANT
BLANK PHE	✘	✘	✓	✘
BLANK ANT	✘	✘	✘	✓
G-PHE	✓	✘	✓	✘
B-PHE	✘	✓	✓	✘
GB-PHE	✓	✓	✓	✘

### Process of dispensing PAHs in the flasks

Microalgae and bacteria were dispensed as described earlier. All culture conditions were also as mentioned above. Experiments were conducted for 30 days without any disturbance in-between. Residual PAHs in the media were measured at one time point only, i.e., after 30 days the residual PAHs were extracted and then analyzed by gas chromatography (GC).

### Quantification of PAHs

After 30 days, the growth was stopped by adding 50 mL ethyl acetate to each flask followed by vigorous extraction. The organic layer containing the residual PAHs was carefully removed and transferred to a dry flask. The extraction was repeated twice with another 50 mL ethyl acetate for all flasks to ensure that all residual PAHs were extracted for GC analysis (Nzila et al., 2021). The combined organic layer was dried using sodium sulfate then concentrated under vacuum to evaporate ethyl acetate. The remaining residue was dissolved in chloroform and transferred to 2 mL GC vials to quantify the remaining PAHs. The following GC method was used. The column oven temperature was set at 130°C and with a hold time of 2 min. Next, a temperature ramps up to 250°C at 11°C/min with a final hold time of 50 min. Post run, a temperature increases up once again to 325°C and hold for 5 min before starting a new run. The total run time for one sample was 63 min approximately. Helium was used as the carrier gas at a flow rate of 1 mL/min. Sample injection volume was 1 μL. Standard samples of PHE and ANT dissolved in chloroform at 5 ppm concentrations were injected using the same GC method to determine their retention time.

Following the GC run, the peak areas obtained were used to calculate the percentage of PAHs removed from the samples by the individual organisms and consortia. The peak area from experimental controls were considered to be 100% remaining, i.e., the peak area from other samples were compared in relation to the control peak area (Patel et al., 2015; Kahla et al., 2021). Table 3 shows the current study compared with previously published work using microalgae-bacteria consortia.

**Table 3:** Studies conducted to determine the potential of microalgae-bacteria consortia in degrading PAHs.

Microalgae-bacteria consortia	Media	Time (days)	PAH biodegradation	References
*G. pectorale* *+* *B. licheniformis*	Modified Bold’s 3 N medium	21	Microalgae-bacteria consortia (99%) Microalgae alone (98%) Bacterial alone (45%)	This study
*Nitzschia* sp. (Benthic diatom) + *Marvita, Erythobacter*, and *Alcaligenes*	f/2 hydroponic culture medium LB rich medium	7	Microalgae-bacteria consortia (88.2%) Microalgae alone (61.4%)	Kahla et al. (2021)
*Chlorella* sp. MM3 + *Rhodococcus* *wratislaviensis* strain 9	Algae maintained on Bold’s basal medium Bacteria maintained on M9 medium Biodegradation experiment done on soil slurry	30	Microalgae-bacteria consortia completely degraded PAHs	Subashchandrabose et al. (2019)
*Selenastrum capricornutum* *+* *Mycobacterium* sp. Strain A1-PYR	Algae maintained on sterilized soil extract medium Bacteria maintained on NT medium	10	Microalgae-bacteria consortia (92.5%) Microalgae alone (85.5%)	Li et al. (2019)
*Chlorella minutissimma*, *Aphanocapsa* sp. + *Citrobacter* sp. SB9, *Pseudomonas aeruginosa* SA3, *Bacillus subtilis* SA7	NT agar, Allen medium and MSM used for bacteria BG 11 used for consortia experiment	10	All inoculants (67.76%) Bacteria inoculants and *C. minutissimma* (92.09%) Bacteria inoculants and *Aphanocapsa* sp. (47.19%)	Omojevwe and Ezekiel (2016)
*Chlorella kessleri* + *Anabaena oryzae*	BG 11 + crude oil (1.0%)	30	Aliphatic compounds disappeared completely when either of the two organisms were cultured mixotrophically in 1% crude oil Microalgae-bacteria consortia Complete removal of aromatic compounds	Hamouda et al. (2016)
*Synechocystis* sp. + *Pseudomonas indoxyladons*, *Bacillus benzoevorans*	Algae grown in BG 11 medium + antibiotics to get axenic culture Consortia experiment done in BG 11 as well BHM supplemented with PYR for bacteria enrichment	16	Consortium could eliminate (94.1%) *Synechocystis* sp. individually was able to reduce a maximum of (36%)	Patel et al. (2015)

MSM, Mineral Salt Media; NT, Nutrients; LB, Luria-Bertani; BG, Blue-Green Medium.

### Analysis of intermediate metabolites during the PHE and ANT degradation

The gas chromatography (GC)- mass spectrometry (MS) analysis is conducted by using an Agilent 5975B MS coupled to 6,890 N GC. The GC separation was performed by using an HP-5 column (30 m length, 0.25 mm I.D., 0.25 μm film thickness) with Helium as the carrier gas. According to the protocol (Nzila et al., 2018) 1 μL Injections of the sample solutions were performed in a splitless mode. The GC oven program increased by 25°C per minute from 60°C to 150°C, then 10°C per minute to reach 260°C (this temperature lasts for 20 min), and afterwards it increased to 270°C (which also lasts for 20 min).

## Results and discussion

### Growth compatibility experiments

**Figure 1:**
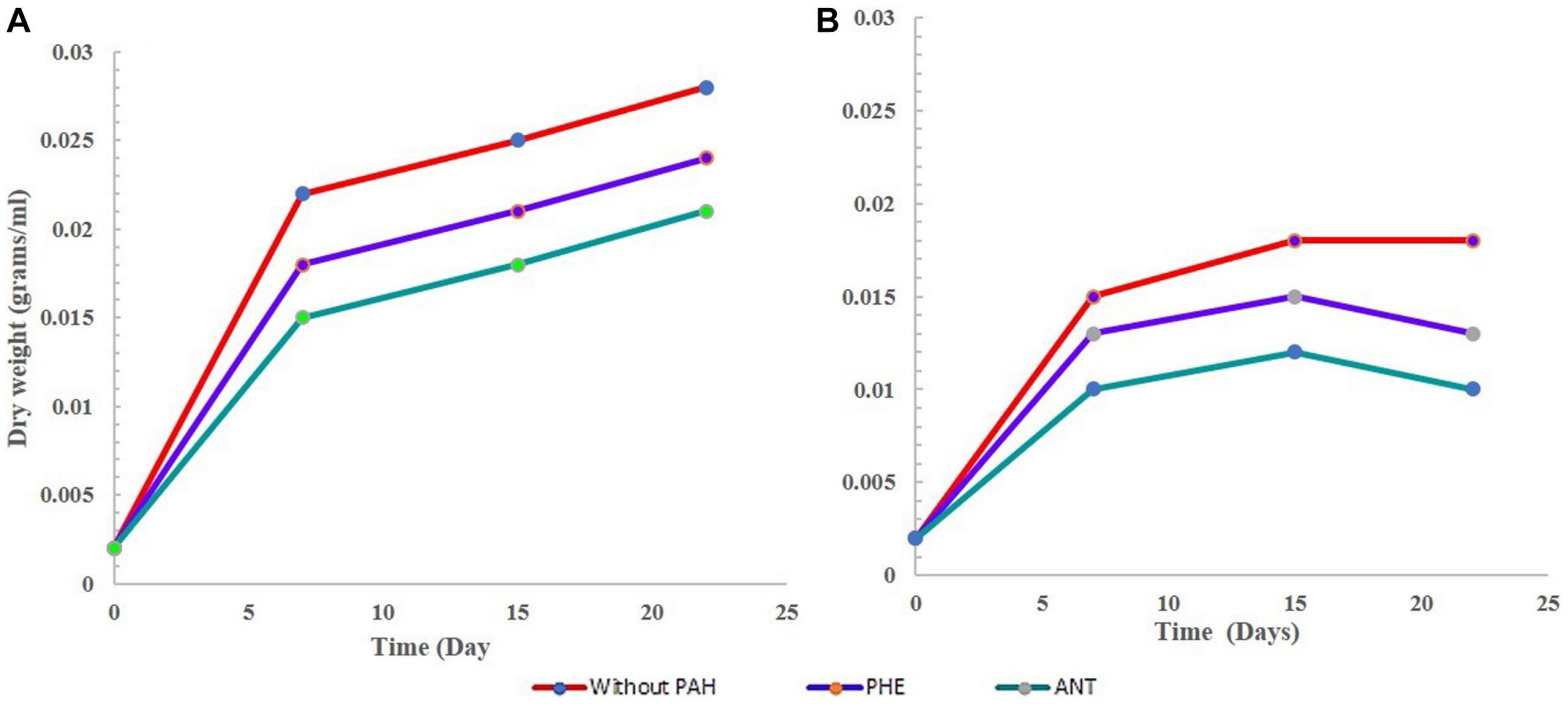
Growth curve of *G. pectorale* without any PHE and ANT. **(A)** Growth curve of *G. pectorale* alone. **(B)** Growth curve for *G. pectorale* in consortia.

Overall, the growth of *G. pectorale* in the experiment performed well and was not hindered by the bacteria. The presence of PHE and ANT seemed to support its growth. *G. pectorale* alone and in consortia but without any PAH, showed a decrease in dry weight after 15 days. However, highest dry weight was observed in *G. pectorale* without any PAH (0.028 g). In the consortia experiment dry weight was slightly lower, i.e., 0.018 g. In both G-PHE and GB-PHE samples, the increase in dry weight of *G. pectorale* was slower (Figures 1A,B) respectively. The final dry weight was also lower than the final dry weights obtained in the presence of PHE and ANT which would have likely slowed down the growth rate of *G. pectorale*. Even visually, when PHE was added to the samples, it initially had a bleaching effect on *G. pectorale*. The green color in all *G. pectorale* samples with PHE was slightly cloudier than the rest of the *G. pectorale* samples. Perhaps, PHE being a three-ringed compound could have contributed to the growth patterns observed. In the consortia sample, the *G. pectorale* was seen to have an even slower growth rate than in the G-PHE sample when measured on day 7. Measured on the 15th day, the dry weights obtained for *G. pectorale* in the G-PHE and GB-PHE samples were 0.028 and 0.025 g/mL ± SD, respectively. In this case, again, why *G. pectorale* behaves in this manner can only be properly answered by further understanding of the interactions between algae and bacterial cells. To speculate, the lower rate of algal growth in the consortia may be due to a lack of CO_2_ which is to some extent contributed by bacteria. Support for this argument can be seen in Figure 1B where it is seen that the bacteria in the GB-PHE sample also decreased since the start of the experiment. Apart from B-PHE and GB-PHE, bacteria in all other samples showed an initial increase in cell count prior to its decrease. In the presence of PHE, the bacteria do not seem to thrive in the media and conditions provided in this study. Therefore, a continuous decrease of bacterial cells in the GB-PHE sample could be responsible for a decline in CO_2_ concentration in the sample which might have led to the slowest growth of *G. pectorale* in this sample. In further studies, it may be worth monitoring the CO_2_ concentrations in the samples to get a clearer picture of the growth kinetics and interactions of the organisms in the consortia.

**Figure 2:**
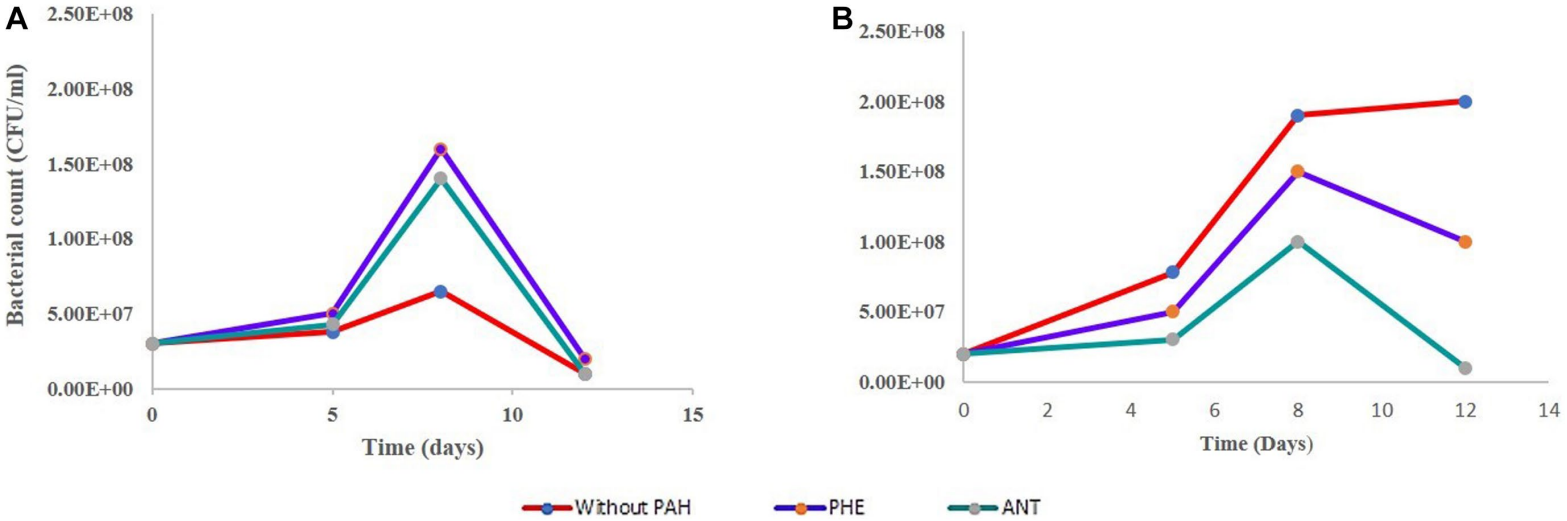
Growth curve of *B. licheniformis* without any PHE and ANT. **(A)** Growth curve of *B. licheniformis* alone. **(B)** Growth curve for *B. licheniformis* in consortia.

In the bacterial sample (B-only), the results are not surprising regarding the growth pattern. The curve in Figure 2A shows a dramatic decrease in cell count measured on day 5 followed by a further decrease. This is expected since the B3N media has no carbon source, and no PAH was added. In its G-only counterpart, we see an increase in dry weight as the cells divide and grow during photosynthesis. The bacteria in the GB-only sample (Figure 2B) shows that within the first 8 days, bacterial cell counts increased due to in the presence of *G. pectorale* in the consortia. When cultured individually without any PAH, it showed a decrease in cell count from the initial 3.0 × 10^7^ CFU/mL to 1 × 10^7^ CFU/mL on the 12th day until finally no growth was observed on the plates (Figure 2A). In the consortia without any PAH, the *B. licheniformis* cell count kept increasing even after 5 days with the highest count reaching up to 3.8 × 10^7^ measured on 5th day until no colonies were observed on the plates. In contrast, the *B. licheniformis* when co-cultured with *G. pectorale* in the presence of PHE (GB-PHE), exhibited a slower rate of decrease with a final count of 2 × 10^7^ CFU/mL on day 12 from 1.6 × 10^8^ CFU/mL on day 8. In the B-ANT and GB-ANT samples, the situation is different with regards to cell count. There was no increase in cell count observed in either sample after initial inoculation. The final cell counts recorded on day 12 were 1 × 10^7^ CFU/mL (Figure 2B). Importantly, *G. pectorale* in samples without any PAH (G-only and GB-only) exhibited an increase in dry weight until 22 days. In the consortia the presence of PHE and ANT in the media did not inhibit the growth of *G. pectorale*. The *B. licheniformis* strain used in this study was previously established to be able to degrade these PAHs (Nzila et al., 2019).

### PAH degradation experiments

**Figure 3:**
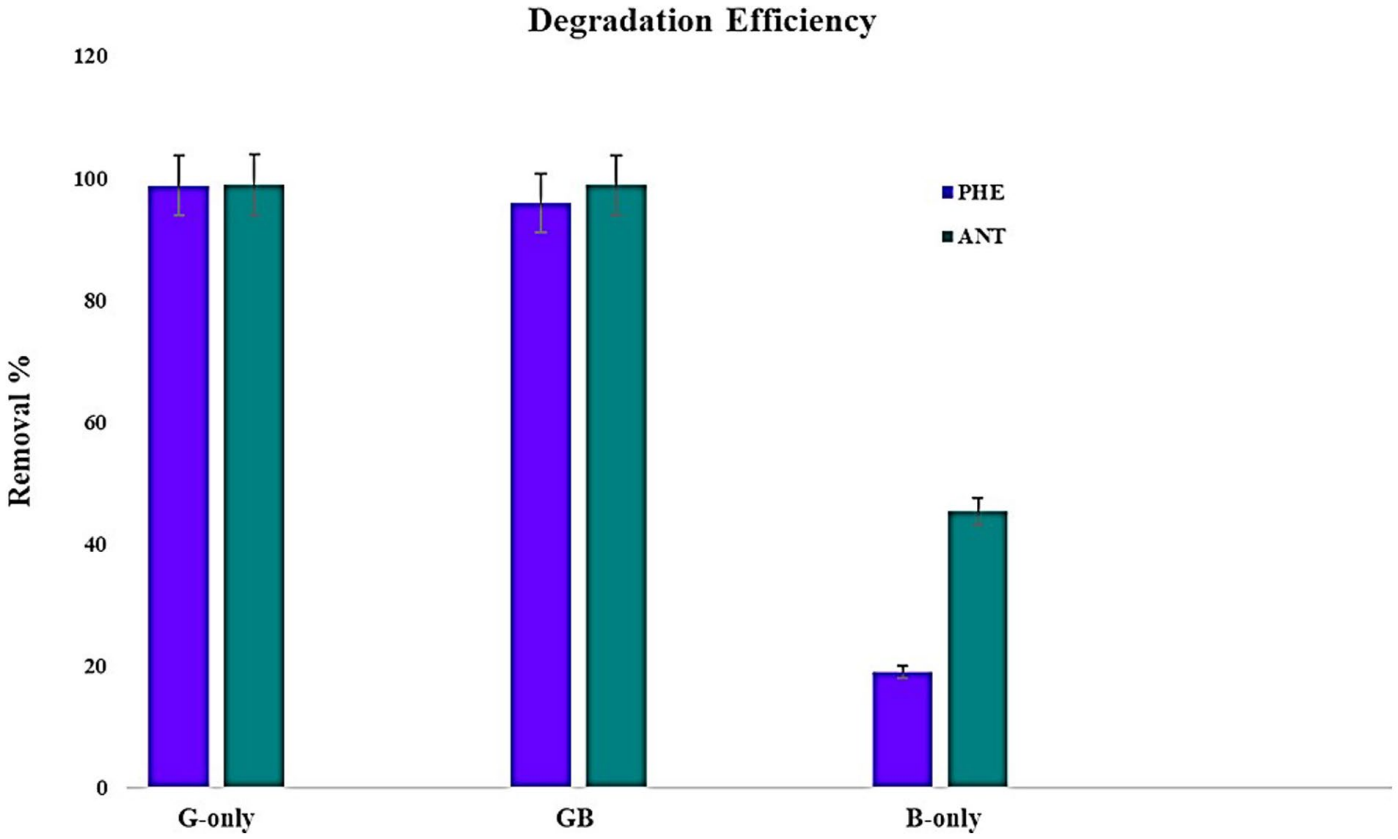
Overview of PHE and ANT degradation by individual strains and in consortia.

To assess the potential of microalgae-bacteria consortia to degrade PAH, experiments were conducted using PAHs with lower molecular weights. Since there are no reports on the ability of *G. pectorale* to degrade PAHs, it was ideal to begin with PHE and ANT. Samples were extracted after 22 days of incubation and subjected to GC-FID. The retention times obtained from GC-FID analysis for pure samples of PHE and ANT at 5 ppm concentration were 13.69 and 15.4 min, respectively. Peak areas obtained for the experimental samples and controls were converted to PAH percentage removed using the formula mentioned earlier. The highest removal of PHE by *G. pectorale* and *B. licheniformis* was 99 and 19%, respectively. The consortia of both strains degraded PHE (96%). Similarly, *G. pectorale* and *B. licheniformis* have removed ANT 99 and 45%, respectively. The consortia of both strains degraded ANT (99%) (Figure 3).

So far there are no reports on the ability of *G. pectorale* to degrade PAHs and the only bioremediation-related data found for *Gonium* sp. was where it was shown to remove various dyes from solution for application in wastewater treatment. In one study it was shown to remove Remazol Brilliant Blue R with the highest rate of 56% among other synthetic dyes tested (Reactive Black 5, Reactive Orange 14 and Reactive Red 120) (Kılıç et al., 2011). Another study reported *Gonium* sp. to be able to remove Reactive Blue 220 with a maximum yield of 54.2% at pH 8 (Boduroğlu et al., 2014). PAH degradation data of *G. pectorale* from this study can be compared to its closest relative *Chlamydomonas* as both of them belong to the order Chlamydomonadales. Literature exists for *Chlamydomonas* and other algae’s ability to degrade various PAHs. For instance, in a previous study, *Scenedesmus obliquus* ES-55 was shown to have the highest PHE degradation of 42% in Bold’s Basal Medium after 56 days incubation (Safonova et al., 2005). In contrast, the *Gonium* sp. in this study removed 71% PHE within 30 days. In the study involving *Scenedesmus obliquus* ES-55, the authors also mention dihydroxy-dihydro-phenanthrene as a byproduct of the degradation process. Therefore, it is crucial that in any future studies with *G. pectorale*, the degradation products are identified, and a degradation pathway established. This is important before any large-scale bioremediation process is designed because the degradation products must also be environmentally safe.

**Figure 4:**
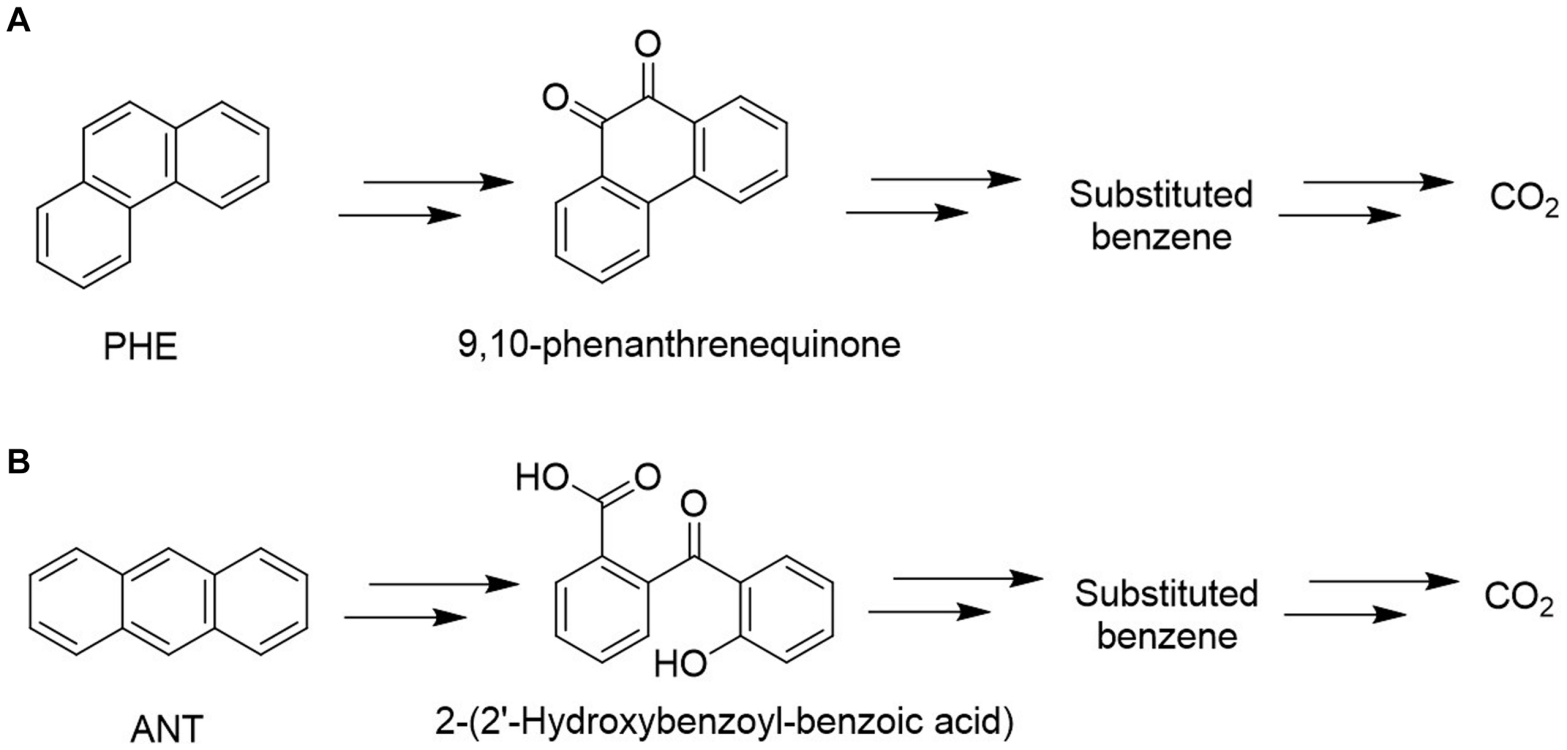
Proposed metabolic pathways for degradation of PHE **(A)** and ANT **(B)** using *Gonium pectorale*.

GC–MS analysis of phenanthrene metabolites revealed that *G. pectorale* produced a metabolite (9,10-Phenanthrenequinone) during PHE degradation (Figure 4). Mass spectrum of 9,10-phenanthrenequinone with a retention time of 20.50 min and contained a molecular ion (M^+^) at m/z 208 and fragment ions 180, 152, 126. Even though the data from NST98 library matches this metabolite spectrum to 9,10-anthracenedione, this is most likely due the similarity in both PHE and ANT chemical structures and thus undergo same fragmentation. Moreover, in a biodegradation study, 9,10-Phenanthrenequinone was identified in *Polyporus* sp. S133 and *Agmenellum quadruplicatum* PR-6 (Narro et al., 1992; Hadibarata and Tachibana, 2010).

Mass spectral characteristics of 2-(2′-hdroxybenzoyl-benzoic acid) was obtained in this study with a retention time: 21.011 (min), m/z 224(M^+^), and base peaks (196,168, and 139). The identity of this metabolite is determined due to similar peaks that were also found for anthracene degradation metabolite produced by Fungi (Jové et al., 2016). The detected metabolites in the degradation of PHE and ANT using *G. pectorale* indicate an inner ring cleavage in both as shown in Figure 4.

### Identification of putative genes in PhycoCosm database

Degradation of PAHs was reported in *Chlamydomonas reinhardtii* and this activity was associated with an increased expression of genes coding for homogentisate 1,2-dioxygenase (HGD) and carboxymethylenebutenolidase, besides ribulose 1,5-bisphosphate carboxylase/oxygenase (RubisCO) and ubiquinol oxidase (Luo et al., 2020). An approach coupling blast and keyword searches into the PhycoCosm database^1^ led to the identification of six putative carboxymethylenebutenolidases and one homogentisatee 1,2-dioxygenase were identified (Supplementary material) in *G. pectorale*.

**Figure 5:**
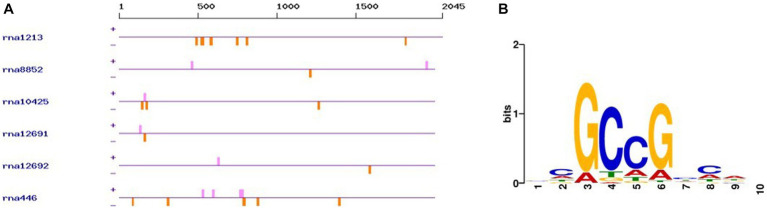
AP2 family transcription factor binding sites in the promoter sequences of the *G. pectorale* carboxymethylenebutenolidases. **(A)** Occurrence of the transcription factor binding sites (orange: negative strand, pink: positive strand); **(B)** Consensus transcription factor binding sequence found.

The promoter analysis of the 6 carboxymethylenebutenolidases (*ca.* 2000 nucleotides upstream the ATG start codon) using PlantPAN 3.0 (Chow et al., 2019) revealed the presence of AP2 superfamily transcription factors binding sites (Figure 5). In this respect, it should be noted that in the green microalga *Auxenochlorella protothecoides*, transcription factors belonging to the AP2 superfamily were associated with T stress and lipid biosynthesis (Xing et al., 2021). Interestingly, in *Chlorella sorokiniana*, pyrene at 230 ppm caused no significant decrease in biomass, but induced lipid biosynthesis by *ca.* 24% (Jaiswal et al., 2021). Hence, it will be worthwhile investigating in the future whether the addition of PAHs to *G. pectorale* growth medium is accompanied by an increased lipid biosynthesis and a concomitant induction of genes encoding transcription factors of the AP2 superfamily and carboxymethylenebutenolidases.

## Conclusion

In this study *G. pectorale* and *B. licheniformis* have been screened for their potential against PAHs (PHE and ANT) bioremediation. *G. pectorale* degraded PHE and ANT (98%) more efficiently as compared to *B. licheniformis* (PHE 19%, ANT 45%). Our findings show that *B. licheniformis* did not inhibit the growth of *G. pectorale* and their combined consortia have shown a slight increase in the degradation of PHE (96%) and ANT (99%). GC–MS analysis has confirmed the metabolites 9,10-phenanthrenequinone and 2-(2′-hydroxybenzoyl)-benzoic acid. Based on our findings, we suggest the bioremediation of PAHs using a consortium approach that seems to be a very attractive alternative to the conventional mechanical and/or other physicochemical methods because it may be the best sustainable and ecofriendly option.

## Data availability statement

The original contributions presented in the study are included in the article/Supplementary material, further inquiries can be directed to the corresponding author.

## Author contributions

MH, AA, SS, DA, AN, and IA designed the study. MH, AA, SS, and MM performed the experiment. AA, GG, KS, MM, and IA analyzed the data. MH, AA, SS, DA, and IA wrote the manuscript. All authors contributed to the article and approved the submitted version.

## Footnotes

^1^ https://phycocosm.jgi.doe.gov/Gonpec1/Gonpec1.home.html
