# Corrigendum: Enhanced biodegradation of phenanthrene and anthracene using a microalgal-bacterial consortium

**DOI:** 10.3389/fmicb.2023.1303835

**Published:** 2023-10-11

**Authors:** Mubasher Zahir Hoque, Abdulrahman Alqahtani, Saravanan Sankaran, Deepak Anand, Musa M. Musa, Alexis Nzila, Gea Guerriero, Khawar Sohail Siddiqui, Irshad Ahmad

**Affiliations:** ^1^Department of Bioengineering, King Fahd University of Petroleum and Minerals, Dhahran, Saudi Arabia; ^2^Department of Chemistry, King Fahd University of Petroleum and Minerals, Dhahran, Saudi Arabia; ^3^Interdisciplinary Research Center for Refining and Advanced Chemicals, King Fahd University of Petroleum and Minerals, Dhahran, Saudi Arabia; ^4^Interdisciplinary Research Center for Membranes and Water Security, King Fahd University of Petroleum and Minerals, Dhahran, Saudi Arabia; ^5^Environmental Research and Innovation (ERIN) Department, Luxembourg Institute of Science and Technology, Hautcharage, Luxembourg; ^6^School of Biotechnology and Biomolecular Sciences (BABS), The University of New South Wales, Sydney, NSW, Australia

**Keywords:** microalgal-bacterial consortium, polycyclic aromatic hydrocarbons, phenanthrene and anthracene, biodegradation genes, petrogenic pollutants, water contaminants, *Gonium pectorale*

In the published article, there was an error in the author list, and author Abdulrahman Alqahtani was erroneously excluded. The corrected author list appears below.

Mubasher Zahir Hoque^1^, Abdulrahman Alqahtani^1^, Saravanan Sankaran^1^, Deepak Anand^1^, Musa M. Musa^2,3^, Alexis Nzila^1,4^, Gea Guerriero^5^, Khawar Sohail Siddiqui^6^ and Irshad Ahmad^1,4*^

As a result of the addition of author AA, the author contributions statement should now be as follows: **“**MH, AA, SS, DA, AN, and IA designed the study. MH, AA, SS, and MM performed the experiment. AA, GG, KS, MM, and IA analyzed the data. MH, AA, SS, DA, and IA wrote the manuscript. All authors contributed to the article and approved the submitted version.”

The citation has been updated as follows: “Hoque MZ, Alqahtani A, Sankaran S, Anand D, Musa MM, Nzila A, Guerriero G, Siddiqui KS and Ahmad I (2023) Enhanced biodegradation of phenanthrene and anthracene using a microalgal-bacterial consortium. *Front. Microbiol*. 14:1227210. doi: 10.3389/fmicb.2023.1227210”.

The copyright statement has been updated as follows: “Copyright © 2023 Hoque, Alqahtani, Sankaran, Anand, Musa, Nzila, Guerriero, Siddiqui and Ahmad. This is an open-access article distributed under the terms of the Creative Commons Attribution License (CC BY). The use, distribution or reproduction in other forums is permitted, provided the original author(s) and the copyright owner(s) are credited and that the original publication in this journal is cited, in accordance with accepted academic practice. No use, distribution or reproduction is permitted which does not comply with these terms.”

The authors apologize for this error and state that this does not change the scientific conclusions of the article in any way. The original article has been updated.

