# Supplementary material for: Enhanced biodegradation of phenanthrene and anthracene using a microalgal-bacterial consortium
Source: Front Microbiol. 2023 Sep 13;14:1227210. doi: 10.3389/fmicb.2023.1227210 (PMC10525690; doi:10.3389/fmicb.2023.1227210)
Supplement: Supplementary file 1 [file Data_Sheet_1.docx]

**TABLE 1** AP2 family transcription factor binding sites in the promoter sequences of the *G. pectorale* carboxymethylenebutenolidases

| **Input Sequence** | **Position** | **Strand** | **Sequence** |
| --- | --- | --- | --- |
| rna1213 | 486 | - | atggCGGCGc |
| rna1213 | 516 | - | cgggCGGCGg |
| rna1213 | 519 | - | gcggCGGCGg |
| rna1213 | 522 | - | gcggCGGCGg |
| rna1213 | 525 | - | gcggCGGCGc |
| rna1213 | 576 | - | gcggCGGCGg |
| rna1213 | 579 | - | gcggCGGCGg |
| rna1213 | 744 | - | agggCGGCGc |
| rna1213 | 802 | - | ctgcCGGCGg |
| rna1213 | 1808 | - | taggCGGCGg |
| rna8852 | 455 | + | gCGCCGacat |
| rna8852 | 1203 | - | ggggCGGCGg |
| rna8852 | 1939 | + | gCGCCGtcag |
| rna10425 | 142 | - | gcggCGGCGc |
| rna10425 | 158 | + | cCGCCGccgc |
| rna10425 | 169 | - | atgcCGGCGc |
| rna10425 | 1258 | - | ctgtCGGCGg |
| rna12691 | 128 | + | gCGCCGacag |
| rna12691 | 157 | - | gtggCGGCGt |
| rna12692 | 625 | + | tCGCCGccaa |
| rna12692 | 1581 | - | agggCGGCGc |
| rna446 | 82 | - | gcggCGGCGc |
| rna446 | 305 | - | atgcCGGCGc |
| rna446 | 524 | + | cCGCCGccct |
| rna446 | 591 | + | cCGCCGccgc |
| rna446 | 762 | + | cCGCCGccgt |
| rna446 | 777 | + | cCGCCGccgc |
| rna446 | 785 | - | gcggCGGCGg |
| rna446 | 788 | - | gcggCGGCGc |
| rna446 | 872 | - | ttggCGGCGg |
| rna446 | 1390 | - | tcggCGGCGg |

**Carboxymethylenebutenolidases (protein sequences) in *G. pectorale***

>jgi|Gonpec1|4981|rna1213

MSSKACCDAGKPVQINYEPKGKFSKAGDLPIYQTGSGERGVVVVPDIFGFGEKQVKHEVAAAVEALRADG

ANKLGCLGFCWAVGVALRAGQDASTFSAVGGAHPALFGHDKELAQKLAVPVVLLPAKGDAPLEPIQHILD

KKPFGSKCVYQRFDDQIHGFVAARGDWSQPDVAAAAGRAIEIMTHFLEKNLA

>jgi|Gonpec1|7933|rna8852

MVRPILLALAIGVACFLAPGANAFGRSLLQECKKNGDRCSLYGPVGQCCDGLLCTMPLECTSKGARSECQ

PECRKQTLGSLVRVNYTSASGTNTPGWEGGNKKKAAVIVMQVRRVVSARRIPTAAEGRVEGWWGVNEAIK

ETALHIAEQGYRVFIPDLYRGRVALNSMEAVGLSSDLNWTRGVDDIVGAANYLRATGSKKVAIVGFCQGG

GMAYCGAQYANVNAAIPFYGLPPNTSICNPLRIPKTVPVQAHYGGNDTSFPMSRVMPAINGMQAAGVNIQ

FNLYPGLPHAFMNGIIPAGRLQMAGGRSNFVYPNETLVTQAFAKMTNFLRLHIRY

>jgi|Gonpec1|8748|rna10425

MESVTFGNGLPGYEVGDKSAPGLIVIQNDIIKDLATTLSKEGFRCLIPDLYKGKIGVDAEEASHLMNNLD

FKNAVDEIKQAVDYLKATGAAKVGVVGFCMGGALSFCAAQHCGVAAAAPFYGTPNPAICQVEKISVPVEA

HFGQLDTYKGFSDVETAKAHEEKIKAGGNKDVVIHIYEKSGHGFFNALAPGGAEALEKHGNPVPPKEELS

AAHERLVAFMKKHLLA

>jgi|Gonpec1|13743|rna12691

MPASQAATVANGATNGVTNGATNGATNGASIGKAAKLCRSPGALCSFYKNDTCCGRLLCTVPTDCGVGGG

LDGCLPVCRNQTVGEFEKIQFPLDVGTAPGFEVGPKTQPAVIVVQIAEQGYRVLVPDLYRGDIALEVTQA

LRLRGELNWNEGIKDVQYAAKYLKSTGSPKVAIVGFCMGGTMAFCGAQYADVDAAIVFYGLVEDHIPCDP

LKIPTSVPVQVQFGGDDSITTVAAVKPVVRAMREEGIDAQLYVYPGLPHAYLNAITPEGRQLRIVMGQDF

RTANETLVTLSFARMISFLRQHIGS

>jgi|Gonpec1|13744|rna12692

MLSVAHRAAPRLMAAVALSLLVIFAMTTAEPAKRTIYKTKNGTRSRVTDGVTDGGTGGVTDAVGEFKKIF

IPSSSGSAPAPAYEAGPKDRPAVLVVQIAEQGYRVLVPDLYRGDVALEVTQALRLEQQLNWTQGIRDVAH

AVQYLKRTGSPKVGIVGFCMGGTMAFCGGQYADVDAVVVLYGLVGDHIPCDPLKIPKDVPVQLQFGDDDG

ITPVSAIEPVAAEMRRAGISTQLYTYPGLPHAYFNALTSEGRQLRIVLGQDFRTANETLVALSFARMASF

LRQHIGS

>jgi|Gonpec1|396|rna446

MLSSDITRPSSARTAAIANDVEAERSSAEVLENFLGWLVANGVKGIGLEDSKVALFASEDGERGLLCEEP

IAAGETVFEIPLRLALTDHPGDDESNQLLYEGAPWSVRLACKLLRHVAAGARSPWASYVQVLPSRVPAPL

EVFGWEDIAGLRYPPAQEALHAADWLRADAFGACGPEATGGLGEEEFRWALSVVHSRTFANAAPGGGVGV

RMLVPLVDMLNHGGDQADPRVRGLAGGAAPTDNVRWDLVAPGPGGGGWVMRVSATAPIAPGQELLLSYGE

RPNDDFFLHYGFVPRANPHDDAVLWPDLEAALEWHHQRFGAGRLSDEEAEPLYRAALEAGLAEQAEERRQ

RAGQAGPGPAPAQQEQDLPAEVLRQLNQVKPELVPVAFGPDRLPGFEIATGPATQPGVVLLQEWWGVTPL

VKQQARLIADRGFRVLVPDLYKGTVGVDMEEAGHLMTSLDFHRAVAEVGQAVEHLRAAGCPKVGITGGCM

GGALSFAAAQHVPGLSAAVPFYGTPAREMPWIEPTGAASGAASGAASLPLVPAPAVQVEKIRIPVQYHTG

MLDPIRGFSDPKNAEEVVGRMRAAGCDVELHLYPATPHSFLNALTPEGVVFLDKWRYGVPPPEQVTLAFD

RMIAFFDKHLKTQ

Homogentisate 1,2-dioxygenase (protein sequence) in *G. pectorale*

>jgi|Gonpec1|6838|rna15131

MATTPAAAPPPSPDAPLKYLPGFGNEFCSEALPGALPQQNNPKTCPYGLYAEQLSGTHFTAPRVANRRSW

LYRIRPSVTHTPFHPIDFPNEALTADFANGAVTPNQLRWRPFPIPTEPVDWVRGLFTLCGTGRAGSKEGF

AIHVYAANRSMESAALANADGDFLIVPQQGALRLKTEFGLLDVAPGEIAVLPRGVRFAVELHCGAARGYV

LETFAGHFALPDLGPIGANGLANPRDFLHPVAWFEPEAERAVSYNVLHKLEGQLFVAAQAFSPFNVVAWH

GNYVPYKYDLARFCPMNSVAFDHPDPSIFTVLTVPSYTPGAPPTADFVVFPPRWLVAEHTFRPPYYHRNV

MSEFMGLIRGQYEAKLDGGFLPGGASLHLCCTPHGPDTATYERAVAGGRGEGGEDGPSRLGHETLAFMFE

TSAIPRVSPAALGCPSVDRDYYKCWVGLRSHFDPTAVPPGVTAPGAATAAVGGKGAGAAASGGVAAGNGV

VKAGKDADRDGGGKANGLAEGLGGLGLS*

Promoter sequences of carboxymethylenebutenolidases

>rna1213

GCAAACGGCCCGAGGAGCGGGAGAGCAACGCAAAGTGCGTGGGCGCGTCGGTGTGCACTGccgctgggagggcgg

ggcgcggcagggtcGACGGAGAGGTTGGGAAAGGGCCAGTGGACCAGTTGACGaaggcccgcagctggcgtaCGG

TGTTTGTAGAGCCTCCGGTCCAGGCCCGGCCCCTAGGCGACCAGGCATGGACTGCCAGCAGGATTTGTTTGGTGCGCGTGCACGGGTTGCATCTGCCGTACAGTGCCGGAGCAACAGGACGGGCGTTGCCAGCGACGCACCTCACCGCGC

AGCAACGCCTAGCGTCCTGGCCGTGTGTTTACCCATGGTTTCGGAACCATGGAAACTTCGCCACCCCTTCTGCGCATGCCCGCCCGACCAGATACCTgctcagctgcgcccgcaAGGTGGGCTGCGTCATCTTCCTGGGCTGGGAAGACGTGGTGGCCGCGAGGCcgaacctgctgctgctgctgatggcggcgctcaTGGTGCTGGACaagaagcgggcggcggcggcggcgcatgaggaggcgggggtagccgcggccgcggtggctggggcagcggcggcggcggtggcggcaggagcagcggtgCCATGATACTACACGGGTCCCAAGAGGCAGCACCTCGAAGAGCCTTGCGTTGCGGTTGCTAGCCGCTATGTTATAGTCATTGACATCCGGAGCGTGGCGATGGGGCTTGCGCAGCGGACTAAGCACCAaaggagcagggcggcgccacggctcATTGTACCTTTTCTTCCTAACGCGCCGTTGCCTTTTGCaactgccggcggcagccgcccatcCTGACTCCCACGCCTCGGCAAACGCTTACGTAtgacgcacctgctgctgaaCCTTGCAAGGCCACTGACGCTTGGACTCCCGTATGTGTGGCAAGTGCATATTTCTTGAACCAACGGGTATGGTTTTATACGTACCGCAtttgcggggggcggccagtAGATAGGAGAAAGAAAAGTGGTAGAGCAGATGCTCCACTCATACTGAATTACAGGCATGAACAA

CTTTTTCCGGGGAGAGGCATTTCCTCATCCAGTTTGCTGTCTGTTTGCGGCGATGTGTGCCCGGTGTGCactgggcggccgcccgtgAGGGCTTGCCGCACCGCTGGTACCGGTATGAATGAATTTCCTTGCATTCCTTTGTTGGGGGTTGGGTCCGTCCTTCCGTCCTTTTCTAGGGAAGGGACGGCCCGTGGCATTGTGCATGCCGGACTGTGTTGTGAAGGAATGAGGCGTCGTACCGCCGATGACGGAGGAGCAGCCACGGGGTGCGGGTCGCATGCGTGGTGGGGCTGCCTCTTGAAGGGTACTTGCTGCGGGTACGCACACGTTGCGACGTGCGACGCTGTCACGCGGGGTGCGTGGCGTGGGTGAAGTATGGGGAGTATCAAGGAATGGGTCAAGTGCATCATGAAggctgcaggaggtgcggTTACGGGTTTCCAGTGCAGTTAGAGTGCACTACCTGTGGCGCCAGGCTAGCTGGGATGCACCGGCGTGCGGCGTCGCCGGGTAttcgcgcggcgccgcagaaGGCCTCGCCACCCTGTGAGGGTCAGCTGTTGCAATTAACCAGCCCGGCTCGCCATATTGATGCTGACCCGTTCCttgacagcagcagctgagcgcCGCATGATAATGCGTTCTGctgagagagggagggaggctcTCAGCGGGCTCGCGAAGAGACGGGGCGCCCGGGAAGAAGCCTCGCCAGGCCGCCGAACTGCAAGAACAGGCCATGATCTCATCATCACATCCCGATAGGCGGCGGAGAACGTCTGGTAAAGGCAGCTAAGCTCAGCTAAGCTCCTGGCTAGCCGAGGTCACCTGAAGGGCTCCGAGACCGTTAAGGTTGCCGACCCGGGGGCCGTTTTTTCCCGTTTCAAGAGCACTGCAAAGGGGCTCCTCACCTGCGATGCGAAGTGCAATTTCGCGCCGCTCCTTCTCGCGATCGCTTTGCACACCTCGCCTCGTTCGCCAGCAGCTTTGCGTTCGCGCA

>rna8852

AGCGGGTCGCCGAGATGCGGCGGCCACAGTGGCCACGGCCTCGCGCGATGTCGGGTCGCACTGGTACAATGCGTACAATGCGGACATGCTGAGGGAAGGCGCCTGAAAAGCAGAAAGCAACACCAGGGGGGGCTACCTGTCcggacatgcacacccgaagggTGCAATGGTTCCAATAGGCCGGCTGGGTacacggcatggcgggcgaaggcaACGAGCgaacgggccctcccggttcGGGCCAGCGGCGAAGTTAGGCGGAAGGCCCCGTAGGCGTGCCCGTAGGCGCCGTGTTCTACGTTGTCACCAATCCATCGGCGAACAGCGATTACTTGACCCGGCCGCCAACCCGGACCGCCAAGGTCCGCGGAACACAGCTGCGGATGCGCGCAGCCGGACGGGTTCCGCAGTTCTATGGGTCCCAAGAATGCCATCGTTGACCCGACGGCATCCCGCGCCGACATGCCCCCGGCCGAGCCGACACCCCACACCGACTCTCCACACGCCTCCGTAGTCCGTACTGCACCACCCACCGAATGGCTACCAGAATCCCAGCCGATACCCCTCACCGacaccccacacgcccccccccccagcacgTGCCCACgtgtcccccaccccctcacccggGTCGGCACACTCGCCGCGGTAGGCtacggccgcgccggcgcacgcTGCCTCACAGGCGTTGCCGTAGGtgctcagccgcctgcccagccgcccgcagacGGGGTCGTAGACGAGGGTACAGACGCAGTCGTTGGCAgatgccgcctgcggctgggccgcgacCAAGCTCACGCCTGCAGTGCACATGTGGGAGTTGCCATCGTCGGGATtgcagctgccaggcggggTATCATCGCACTCAGTATCGAACTCAGAACCCAACAGACAAAAAGAGGTACTAGTGGCGCGCATGACCTTCACGGCGGACCCTtcacgccccgccctacccccccccctgccctcggTCAAGGTACAGGCGGGGGACGGATCCagaggggagggccggggcgagcgggcggggagcatCACGCACCCCAGCTGCCCTATGTAccagcggccggccaggcAGACGAGCGGATGGAGAGAGCGCATTGAGCTGTCTCAAGTACGGTCGAGCGGCTATATGtctggcgtgcgtgcgggcgtgcgttTGGGAACCGGCAAGGCGGCAACACGACAACACGGCaacgcggggggcggcgggcgtgccaaAACATGTAGCGCCGGTTGAGCCGGGAGGGTTTTGCTTCAGGGGTGTATCGGCCGGTCCGCtccgcaggcaggggcagcgctggcgcgcaCGCTCTGTTGGAGATTGGATTAGTACACAAGCATGCTGGATTGCCGGTCTTACCCGCAAGCGAAAGCAGCAGGaaagccgctgccagcgcgctcATATGTGTCGCCCGGGGCGCTGGTTCCATTTGAGCGACTCCTCGCGACTTGTTCCGAAACGAGGACGCGGTAACGAAGCTCTTGGGTATATATACCGTATATTTGGGGCAACTCGTTGCAAAATGGAAACGCGTCTCGCCTCACGTTACATACCTGGACCGTTGTGCGGAAACTCGCAAATTCATTATCCCCTCTCCGACCGCGCCCCGataccgacccgtgggccaaggcgggccggccctggtgccggccgccgctgccatggcgaACGCATGGCGTGATCTCATGAGCAAAACTGAGCAACTTCGGCTTCGCATTTGCGCCCCTGCCGTGTGAAGCATGAGTGCATGACCAAGGAATGAACAGTTAGGGTTCCTTAACTGTAACTCCCCGTGGCCGGACACATGTTGTTTATGTGTCTTGCAGGATTACGCCAGGTTCTCAGCGGTGAGAAGCGCGAATTCCTCTCTTTGCGGTTGGTCAGACATAACGGTCGAACCTGTCAACGTGTGGTAGTAGATTCGTGCGCATGtcgcgccgtcagcgcccctATATTGTAATTTTCCAATTTGTAAACATTTGCATCAATTGCGAAA

>rna10425

TGGACGCGGATGAGTCCGGCTGTGCCGCGCTCGTTCACCACCAGGCTGATGGTAATGTCCCGAGAAGCCATCATGGCCggggcgcctcggccggctgccccggcagcggacacagcgcccgccgcatccgcatcaccgccagcggcggcgccggaacccgccgccgctatgccggcgccggtccccTGGCCCTtggtggcccgcagcgcgttATCGATGAGCTCAGCCAGCGCAAAGGCAAAggggtggtggccgcgggccgAGAAGTACTCGTAGTCTCCGGCCATGGTCAGCGTCTTTGGATGGGGCACGAAGCTAATGCGCTCCTGCAAGGAGCATGCAGGAATAATGTTGTACACATTATGCCTCCCGTAGCTGACCTAAATGTGGAAGCCGAATAGCATGCAATCTGCCGTGCTCTGGTTTGCAAGAGCCCAGGCGCCGGCTAAGCTACTGCCCGGATCATCCCGGGAGAACCTAGGCATGCGGCTCACAGGCGGAGCTGTACGACCCGTGTCCGTGCCCCAATGAAGGACCAGCTACCTCTCCATGCAGCACAATACATACTAAAGTTGTATACCGTCCTTTGATCTTTCTTGATCTCTCCCCCCTACGCTCTTAACGCCGCAGCCTTAGTACTCCGGCACACCTTTGCCGGTGGTTTGTACCCGCTGTCCTGCAGGGTTGCGTTGCTGGCAGGCGGCATCGATACAGggccggcgacgctgctggggcgcggcggccgagaaAGGCTCTTGCTGCGCCCGGCCGTTCCCggaggcagcgacgccggggccatggagccgcccgggccgcccgtgGAGACAAACAGCTGGTCGCCGGCTCGAAGCTTCCGCGCGGCCACGTCCGAATCGATGAcgcgggagctggaggacaTAAGAGAGAAGtcctgcggggggtggggaaagCCTTTACGGTAACCGACGCCCGTAACACAAGCAAGGCAGTTGCGACGTGCAGGCCTACATGGCCGCTCTCAAGCCCTCCCcagcgcgcgcgtggggcCAAGAGGATGGACGGGCCGGGTGCCGGAGTGATGTCTGCAGCCGTGGCCCCAGCGACCCGAGACCATGGCCGCCCGCACTCAAGGCTCTGGGCGTCCCTTCGGGAATTTGGGGACACGAACCAGGGGGCTAGGGACACCAAACACACCTGCGCGTCCAGCTCTGGCTTGAAGGCCTGGCGCACCGCTTCCGTGAGGGACTTTAGGTCAGGGAAGGGTGTGCATAGGCGCGtctgtcggcggccgccaagacCATCCTTGAGCAGCACCGTGACGAACTCTGGCATGGCGGAGGGAGGATTCCGCTCAAGTGCGCTGGTGGTAGTGTTACACGTCACACGTAGCCCCTACGATTCACAGTGGCCCAGTTGCGCGGCTTCCAGTACTCGTGTTGAGCACAGACGGCATGCCCGTCGCGGCGTGCCCTTGCGAAGCGCACAACACACGCACCTGACGTACTGCTCATACTTCTCCAACTAGGCAAGCTCCTGGATGCGCCCTGGCAACGTCCCCGCGCAGGCTGTCCTCGTCTGCGGCTGAGCCTCCGAGCGCCACATGCACCAGTTAGTAAATGACATGTGAAGCATCTATAGATGATCAACCATCAACACTTTGTAAGGCACATCCGGAAGCGCTGTGGTTCAGTTGTGGCAGAAGCGCATGATGACGCATTTGTGTCGATTCATTGCGGCGAGCCATTTCAGCATTGGTGCAGTTGCGTTCGCCGCGACGAGAGCTGCCAGTGGAACCGGAATCCAAGAGAAACATCTACCCTACCAGGCACCCACATGCCCGCACTCCGTTACCCTTTACCCCGCCATAAAGTCCACTCACGACTCCTGCGACGTccagcacgcagcgctgcccgcctctcACAAGTAAGCATCCTTTTACGTGCCATTCCTTGACACGCCTTCAATGAATGCATGCCAGTAAGCATTACTTATGCCCGTCCTTCTGGAACCGAACTTCAAGG

>rna12691

GGCCGCGTTCAAGGTTTCGACAAATTGGTGACAGCTGGTG

GTATTCGTGCGCTGGAAGAGCCTTGGGTCTCCCCGGTTTCAGGAGCAGCGCCCTCTGCAG

CCCTCCTGAGCGTGTGGCCCGGTAACGGCGCCGACAGCTCTGGCGGtaggctggcggtgg

cggcgtgggccgcaACGGAGGTGGGCGCTGCCGTGACGCACGGCGGAGCTTTTCCCAGGC

CAGCAAGCAGCCAGGCAAAAGGAGTTTACATTTCGTCTCCCTTCGTGCAACTTCTCGACA

CGCCTTTGACGTCTATTGACATCCTCACCAGTTACAGTCAATATGTACGATACCGGACAT

GGAGCGAGTGGTTGGAGAGACAGCGTGTGTCAAGCGTGCGGGTAAGGGAAGCGTGTCCGG

CAGGTCACGCTGGGTCACCAGTCTTGTCCCAAACTCAAGTCACGCAACCTTCCTCGGCAG

CCGCATTTCCGTTTTTTTTTTCCGTGTCGCCGGCTCATACTCCGATTCCAGCCCGCTTGC

CACGCACATTACTTGGCACTACAGGTCCTAACACATGCTACGTTTAAGGTGCAAAGCCCT

TTTTGGCCCCCCTACCGTATTCCCATGTTGGTGGTCAACCTGCATAATAGCAAAGACTAG

GATAAGCCTAGTTGTTTATGTATGATTATTGTCTATATCCCCAATAAACAAGTCAGGCCC

CTTTCattcaaccccccccccctcacatgTAGGTCTGTGCGTGCACCGCCGCTACGTAGG

CGTTGCACGCAAAGATAAGATGGTAAGATCTAAGATGGTCTGCTGGTCTcgccggacggg

gcgggagggcccttCAGCCCTTGTTATGCCACCTTGCCTAGTACCACTCCTCTACAATGC

GGGTAACGTGCCAACATATGTCGGTTCGTGGAGGTGCTCAATGGGTCTGCTACGATTTTA

TATACAAGTGGATGGCATGTTTCGGAACTTTATTTTGCGTCCAGTAAACCCTCGCACCGC

GCCTCTTGTGTCCGGTGCGTGCGTTACGCGGGGTGCAAGCGATGCTTGACACGGACTGTT

TGACTAGGACCCTTGTCTACCAGGGACTTCCCCATTTTCGTGTTTTGAGACAAGTGGCTG

TGAGTTGCGGCTCTTGAGGTCCTCTCGTGCCCacgctgggcgggcgcggctgcgcgtcgT

GTCCAGCcaagccgcgggcgccggaccGCTCGGCCGTCTCACCTCTAGCCAACCTTCATC

CCGCAGTAGTTGGACCCCATGTTGACCAATGCATGCGCATGCGCGCCAATGAAATGCATG

CTCTATGATTACTGTCTTAGAAGCCAACTGCGGCCAATCTATTTCCCCGTGGTAGTCGTG

GCGGGATTGTACCGTCCTTTGACCCCctagcgacccacgggtcggcggcgacggagcaaA

GCCGCCCTTACCCcccgccgcagaggcgcaccgagggagcggggctgtAGAATCCAATAC

ACGCGTCGCGAAGCAGAAGGCGTGTACCTGCCCCTACAGCCTCCCAGTTCGCGCAAGGTA

GCGAACGACCGTACCGTAAAAACCGCTGCTAGCTGCCTGCCCTGCACATCAGATACCAAG

TATGAACCACGAGGCCATTGCGCATGAGGTGTCAGGCGGTCCGACGCCACGCCACCCCTC

GAGTTGAACGGTTGTGATACCCACATATCTCTCGGGGAAACGATACGTTGGGCGGCTCCT

CGGCCTCGCTTTCGTACTCGTCGGTCGCTCAGAATCCACATGAGAGTCGAAAGGGGCGTT

TCAGTATTTTGTGGGGAGCCCGACTGGTCAAATCGGGCACACGCTTAGCCGTCCGGGTCC

GGCCTATTCAACGCCCGAGCAACAGAGGTGGTCGCACCCATAAAAAGGCAGAGACCGTTC

GTAGAGCGTGTGTGCGTTTCGAGGGATGGCGCTCCCAGTGCGCCGCGTGACGCCGGTcgt

cgcgctggcgctggcggccctcctaGCTGCGTTCGTGGCG

>rna12692

CCTGCgctccttgccgcccacctacctgccctccttcccctctcctcttcccctctccct

accctctccccttcccctgttcctcccgcccctcctccctttcCCTCAAATGCGCGTACG

AGTCCAGATACCCGGCATATAATGCCTCCCACCTTCCGTTAcccttgccctccctccctg

tgtccccccatccctgcccctCATGTCGTCTCGTGTGTCCAGCTaccccggcctgccgca

cgcGTACCTGAACGCCATCACCCCGGAGGGGCGCCAGCTTCGGATAGGTAAGAGCGTTGG

GCGCGCGGGGTGAtttgcgtacggcagcaccgaCATACGGTTCTCCCGGTCTACGGTACC

GCCTGCTACTGTGTCCGCGGGCGTACAGCGCGTACTGCATGCCGTAATCTTGTAGGGGAT

GGCGTGCCCTCCCCTGGGCCGTAGCTGGGCCGGTTCAGTACCAGGGAAGTGCCTTAGGAC

GCGAATTGAGCACCACATGCTCGGCGAGGGAAGAGCGAGAAGTCTTGAGCGGGCACGTCC

AGGTCACCCGCCGTCGCAATGCGGTTCCCGCCCTGTGTACCGTACATCCATGTTTTTTCG

GTCCGCTAGATTATGAGCGTCCTGTCGCCGCCAAAGCGTTCACTTGCGCATTCTCTGCCT

ACCTGGCCCCATCCTCGTTCTTCCCACCGTGCCTCCACCAACCCCCAGTCATGGGCCAGG

ATTTCCGCACCGCCAACGAGACCCTGGTGACGCTGTCCTTCGCCCGCATGATTAGCTTCC

TGCGCCAGCACATCGGAAGCTAGGGAGGAGGAGCGGTGGAGGGATCTGGGTGTGAGGCGG

AGAGGGGACAGAGAGGGGATCAAGAAAGGACAGCGGAGATGCATTGTACGGCACTGTCGT

GTGCCCTCTCAGCAGTGGATGGCCTCTCCGTGCTCTGCGCTTCAATGCACGACCGTTCGC

GTGCATGCTCGTAGCTTGTATCAGGGCCCATTACGGCAGT

ACTCGTCCCTTGGTGGGGACGCAACCTGAGATGCGCCCCTGCGCGAACCTTGCTGTGTGT

TGGCCAAATGGGAAAGTACGACGCGTCGTGCTGCGCGTCCTCGGTTGCTGACCTTGCTGT

TGTTCTCCAAGCGAGCTTGCTGACTCGTGTGGTTCGTGGGAGGACGGTACCGCAATGCTT

GCACgcagacggcggtggcggagacggTAGACGCACGCAGACTAGGTAAGAATACTGAGT

GCTTCGGGAGCATGGCCCTGGAGGTGTGGCCCCCTCATTGCAGCAGTATCACTGCTCTGG

GGCAGACACCATGCTGCCTCTTGTGTATGCTGCGACTTGTGTATGTATACTAGCGTTTAT

TTGGTTTCTGGTAAGTTCTTCATTTATGGTTGACGCCATCGTCTTCCTGCCCTTGCGTGC

CTGGCATTGCACCGCGGTTTGCACCTTGCCAACTTCAGGCGCTTTCCTCCCGCGTTGACT

TCGACTCTGCTTTGTTTGGGTAGGTGGGGAGGCATGTAACGACAATCAGAACGCAATCAG

AAACTCCTGCCCCGCGTGTAATGGCGTTGGGACGACgatgagggcggcgcgggtcctGTT

GTGGGGGCTgcacgacccatgggtcagagTAGGCAGCGGGCCGTTGAGGTTATCCTGCTG

AGGATGGCCACGTTCGCATGCGAGGCGAGAAGGCAATCATGGCTTATTGTTTGCTTGAAT

TTAGCTTGCCAAGCGAACTTCGGGGTTTGTTCGACGTGGAATCTCGCTCGGAACCTTGGA

CCAACCACGCTCTCGTCGAGGCCACCGCCCGACCCGTTGGGGGCTTCGATAGCTCGTCGA

CTCAGTACGGCAGCTGAATAGCTAGAACCATTGCCCTATGAAACAGAAGACGCCTCTCAC

CGATCTACAATTAGAACCTGCAGCTCAGCCCGCTGGGGCCCGGTCCGTTGGAAAGCCATC

CCCACACAGATCCTTAGCGCTTCCCGCTAAACAATTGAAA

>rna446

cgtgcccgcacccggcctccctgccgccgctgccgcagccgcagccgcagccaggtccGTGAACTGCGGCTCGGTccccatgcggcggcgctggttgcggcggggcccgtccagcgccacccaggccacctgcacgccggagggtggggctcggcctgtacggcagttatgcagctggtgctgctgctgctgctgatgtggTGACGGTACTGTCGGCTGTGGAAGCTGCGAgggaggctgtggcggtggggccagcCCGACTGTATGCGATGAGCCAGTTGCGGCTTGAGGTGCTGCGGGCAccatgccggcgccagctgcgtgggcggggaaggcccccagcgccgcagccggctcgtGCGGCTTGCCGACCGTCACCAGCTGATGAGGATGAGCCTGAGCGCCGCTTgtcgtgcgctgccgccgtcgtagCCGTACAATGACGTACAccgtgacggccgcggcgactGCGACGGTCCCTGCGCACACGGCGATgagggtggcgaggagggaaaccgccaggccgccgccctcctgcggcggccccggcggaagcggccctggcggtatcggcggcacccggcgcgcctccgccgccgcggcctccagcgccattAGACCAGTGAAGGGCTGACCTACCTCCCAGCTCAGCTCTAGTTCATTGGCACCAGTGCTGAATCCCAGGTTGCCGGCACTTtcggggccgctgggtgggGTGCCCTCTGCCGAGCCCACGTAGCGACTAAAGCCtgtggtgcggcccgccgccgtgcctgccgccgccgcggcggcggcgctggcgttcgacgccagcggcgccgtcgtgctcagctgctgcaccccgaTGCCCTCACCGGTCGTGCTGACcttgttggcggcggggcggccagcgacgccggctTTGCCGGGcacgctggggcccgcggggggcggcgacggcggcagcgcgacgcaGAGGAAGGGCGCCACGAGGCCGGGGGTGTCGCAGGGGaaggccgccagcagggctcccgaccgatgcccgccgctgcaccccagcagccgccctggcctAACCTGCCACTGGAAGCCGCCtgatcctgctgctgcagctgctcctcgggCGGTGTCTGCGCCGACTGACCACCCAGATCGGCCAAGCCGGGGGGACCACTAGGTGGTATGGGACCCTCCTCCGGCCACAAGCCCGGGAGCGATCCCGGGCACACGCGCCGGACGCCCGCAAGGCCCCCCAAGTGTTgagccgaggcggagggcaggctTGCCAGCCACACAAGCTGACGGATCGGCGCCGAAGCCGCGCTGTCGTaaccgctggctgcggcttgGCCCTGATCAGGAGCCGCCGCGGTCCCCAGGTGCGttgcggtgccgctgcctgggcttgcatcggcggcggaggagggggggtcgGCCCTGTCCTCCAGGGGGCTGAGGATAGTCTGCGCCAGCCATAGCACTAAGTCGGGGTGaagggccgaggcgggggccagggaggtGGAGGTCATCTCGCACCACCGCGTCGCGCCGGCGAAGCTCATGGGCCACGGGTTGTACGTATACTGCGCACCGCTGCTGATATAGCTCTGGACTTGGCGGACGTTGCCTCCTATGGTTATGGCATCATGCGGAAGGAGGAAGAAAGAATGAGTGATGGCGGAGTGAACAATGGACGCGGGACGTGGGAACCGGTTAGACTGGGAAACGGATTTACGGACGCGCATGAATGAAATTGTAGCGGAACGTAAGGGAAAAGTAGATTGCAGGTATGGTTGTGGTTGTCCCGTCGCACCGGTCGGTCACGTCAGGTTAGGTCATGGCATGGCGAGCGTGCGACGAGGGATTGGCCCCACTTCAGCTTACCGAGATTTAACGCTTGCACTGAAGTGGTCATTATGAACAAATGTGCTGCTGCATGTACAAATGCCCGCAATGGGAGCGGCATGGCGCTTTTGTGTTTGGACCAGCAGCTCTCCTCTAGCCTACAATGTACGCTCACA
